# Cultural values and HIV prevention intentions: integrating Hofstede's dimensions and the theory of planned behavior in Amazonian Indigenous communities

**DOI:** 10.3389/fpsyg.2026.1789791

**Published:** 2026-08-28

**Authors:** Angelica María Carrasco Rituay, River Chávez Santos, Omer Cruz Caro, Polito Michael Huayama Sopla, Roger Guevara Gonas

**Affiliations:** 1 Instituto de Investigación en Negocios Agropecuarios – INNA, Universidad Nacional Toribio Rodríguez de Mendoza de Amazonas, Chachapoyas, Peru; 2 Facultad de Educación y Ciencias de la Comunicación – FECICO, Universidad Nacional Toribio Rodríguez de Mendoza de Amazonas, Chachapoyas, Peru; 3 Grupo de Investigación Comunidad de Responsabilidad Educativa de Amazonas, Universidad Nacional Toribio Rodríguez de Mendoza de Amazonas, Chachapoyas, Peru

**Keywords:** Awajún and Wampis, culture, Hofstede's dimensions of national culture, Indigenous health, prevention

## Abstract

HIV prevention remains a priority challenge in Indigenous communities, where conventional public health strategies often fall short by failing to adequately incorporate the cultural factors associated with preventive behavior. This study addresses this gap by integrating Hofstede's individual cultural dimensions with the Theory of Planned Behavior (TPB) to analyze HIV prevention intentions in Awajún and Wampis native communities of the Peruvian Amazon. Using a quantitative structural equation modeling design, the relationship between cultural values on attitudes, subjective norms, and preventive behavior intentions was examined. The results show that cultural dimensions are associated with the TPB constructs in different ways. Long-term orientation and masculinity showed positive and significant associations with attitudes toward HIV prevention, although uncertainty aversion exhibited a negative effect, and indulgence did not show a significant relationship. At the normative level, power distance and indulgence emerged as significant positive predictors of subjective norms, while collectivism did not show a direct effect. Consistent with the TPB, attitudes, subjective norms, and perceived behavioral control were significant positive predictors of the intention to prevent HIV, with perceived behavioral control showing the strongest predictive effect. These findings provide novel empirical evidence on the differential role of individual cultural values within the TPB framework and highlight the importance of considering cultural orientations and perceived capabilities when designing HIV prevention strategies in indigenous contexts.

## 1 Introduction

Indigenous knowledge constitutes a system of knowledge deeply rooted in the cultural, social, and environmental contexts of indigenous peoples (Ijatuyi et al., 2025). It encompasses practices related to land use, ecosystem management, traditional medicine, sustainable agriculture, and indigenous healing systems, integrating environmental, productive, and health dimensions (Jessen et al., 2022; Whyte, 2018). In conceptual terms, indigenous knowledge refers to long-standing traditions and an intimate knowledge of local ecosystems, biodiversity, and social dynamics, developed by indigenous peoples and local communities over time (Berkes, 2009). Far from being informal or pre-scientific knowledge, indigenous knowledge has been recognized as a dynamic and sophisticated system, based on processes of systematic observation, empirical experimentation, and continuous adaptation (Berkes, 2017).

The traditional ecological practices and knowledge of indigenous peoples develop through generations of empirical observation, constant interaction with the environment, and progressive adaptation to environmental changes (Manningtyas and Furuya, 2022). For example, traditional agricultural systems developed in Andean and African regions employ complex soil and crop rotation techniques adapted to specific microclimates, ensuring ecological sustainability and food security (Ijatuyi et al., 2025). This evidence reinforces the understanding of indigenous knowledge not only as a body of knowledge, but as a cultural framework that guides individual and collective decision-making, including those related to health, illness, and prevention.

In the field of health, worldviews, belief systems, and cultural values specific to each community are linked torisk perception, the interpretation of disease etiology, and the legitimacy attributed to biomedical interventions (Chance et al., 2025). Therefore, preventive behaviors cannot be understood exclusively from a biomedical or informational perspective, but must be analyzed in relation to the cultural frameworks that structure behavior (Hofstede, 2001). In indigenous contexts, these cultural representations may associate illness with transgressions of social norms, spiritual imbalances, or non-biomedical causes, conditioning the acceptance or rejection of specific preventive practices (Flores, 2020).

At a structural level, indigenous peoples face persistent disadvantages in accessing health services (Carrillo-Larco et al., 2022). In the Americas, approximately 21% of the population lacks access to health services due to geographical barriers, a situation that disproportionately affects indigenous populations (Cassiani, 2014). These groups exhibit worse health indicators compared to the non-indigenous population, including a higher prevalence of infectious diseases such as tuberculosis, HIV/AIDS and other sexually transmitted infections, as well as higher maternal and infant mortality rates (Singer et al., 2017). Peru, home to the second largest indigenous population and the third largest number of indigenous peoples in Latin America, has 55 indigenous peoples organized into recognized communities, which have limited access to medical care and worse health conditions than the general population (Hernández-Vásquez et al., 2022).

In Peru, the Sectoral Policy on Intercultural Health was approved in 2016 with the objective of regulating intercultural health actions and guaranteeing health care as a human right; however, its implementation has been partial and the effective results remain limited (Carrillo-Larco et al., 2022). Only a third of indigenous communities in the Amazon have access to a health post, and in Puno, located in the southern Andean region, the majority of the population did not have access to screening tests for non-communicable diseases (Calderón et al., 2019). Furthermore, the COVID-19 pandemic has highlighted the exclusion and neglect of Indigenous health in Peru, evidenced by a lack of preparedness to provide adequate and timely diagnostic testing, healthcare centers, and specialized treatment for severe cases. Data availability to understand the effects of the pandemic on Indigenous peoples in the Amazon and Andean regions has been virtually nonexistent, especially during the 1st year of the pandemic, and some reports indicate that Indigenous peoples in the Amazon experienced a higher mortality rate compared to the general population (Zapata, 2020).

In this context of structural inequality, the Human Immunodeficiency Virus (HIV) represents a particular public health challenge. Globally, HIV continues to be one of the major health problems, with approximately 40.8 million people living with the virus at the end of 2024 (Afzal et al., 2025; UNAIDS, 2025). Although prevention strategies have progressed, their impact has been uneven across populations and territories. In Amazonian indigenous communities, HIV prevalence rates have been documented that far exceed national averages, as in the Warao (Venezuela), Chayahuita (Peru), and Wayúu (Colombia) peoples, where prevalences above 5% have been recorded (Russell et al., 2019). Prevention strategies have progressed, but their impact has been uneven across populations and territories. In Amazonian indigenous communities, HIV prevalence rates have been documented that far exceed national averages, as in the Warao (Venezuela), Chayahuita (Peru), and Wayúu (Colombia) peoples, where prevalences above 5% have been recorded (Russell et al., 2019).

In Peru, although the national prevalence of HIV is estimated at 0.3% (Ministerio de Salud del Perú., 2024), the Awajún and Wampís indigenous peoples face a particularly critical situation. These ethnic groups, which represent approximately 35.8% of the national indigenous population, account for more than 70% of the cases reported in the Amazonas region, demonstrating a marked epidemic concentration (Valenzuela-Oré et al., 2023). This disparity cannot be explained solely by structural factors such as poverty, migration, educational gaps, or limited access to health services, but also responds to sociocultural determinants that condition the adoption of preventive behaviors (Benzaken et al., 2017; Russell et al., 2019).

Cultural dimensions are related to decisions related to HIV prevention, including condom use, acceptance of diagnostic tests, and adherence to preventive interventions (Zidkova et al., 2025). Despite recommendations made by international organizations such as the Joint United Nations Programme on HIV/AIDS and thePan American Health Organization, for the development of culturally relevant interventions (Russell et al., 2019), however, studies in Indigenous communities have primarily focused on other areas of health, addressing culture as a general contextual determinant. In the field of HIV, available studies have prioritized knowledge, prevalence, barriers to access, or risky sexual practices, without integrating theoretical cultural models or examining the association between cultural dimensions on preventive intentions as an antecedent of behavior (Braley et al., 2023; Cáceres et al., 2023).

From a theoretical perspective, incorporating cultural dimensions into the analysis of preventive behaviors against HIV contributes to the development of an emerging line of research that recognizes the role of sociocultural factors as determinants of health in vulnerable populations (Valenzuela-Oré et al., 2023). From an applied perspective, understanding these mechanisms is fundamental for the design of contextualized preventive interventions, since evidence in global health underlines that preventive strategies must be aligned with local value systems and practices to ensure their acceptability and effectiveness (Zidkova et al., 2025). Within this framework, the study aims to determine the predictive role of cultural dimensions, operationalized from Hofstede's model, on the intention to adopt HIV prevention behaviors in the Awajún and Wampís indigenous communities of the Amazonas region, seeking to generate scientific evidence that contributes to the design of culturally adapted public health interventions, aimed at reducing vulnerability to HIV in these populations and in other similar sociocultural contexts.

## 2 Literature review

### 2.1 Hofstede dimensions

Hofstede (2001) proposed a model of cultural values that characterizes societies along several quantifiable dimensions. He originally identified four dimensions: power distance, individualism-collectivism, masculinity-femininity, and uncertainty avoidance; he later added two more: long-term orientation and indulgence vs. restraint. These six cultural dimensions describe differences in how people deal with power inequality, the relationship between the individual and the collective, gender roles, uncertainty aversion, time orientation, and desire (Matus, 2021). Hofstede's model has become established as a dominant framework for comparing cultures across different countries, widely used to contextualize cultural variations in scientific and social research over the past few decades (Kole, 2025).

These cultural dimensions are not isolated traits but standardized constructs that capture shared value orientations at the societal level. They function as analytical lenses that allow researchers to systematically compare how cultural contexts relate to individual cognition, social norms, and behavioral tendencies. In this sense, each dimension represents a continuum along which societies vary, influencing how individuals interpret authority, social expectations, risk, and interpersonal relationships. By operationalizing culture into measurable dimensions, this framework enables empirical examination of how deeply embedded cultural values are associated with attitudes and intentions in specific domains, such as health-related behaviors (Hofstede, 2001).

However, Hofstede's scores are derived from national averages, which can mask significant individual variations within each culture. While this approach allows for the identification of general trends, it does not imply that all members of a nation share a homogeneous cultural profile. In this regard, instruments have been developed to measure cultural values at the individual level, allowing for a more accurate capture of intra-cultural variability and avoiding oversimplified inferences based solely on general averages (Dhingra et al., 2024). In particular, Yoo et al. (2011) created the Cultural Values Scale (CVSCALE), which assesses Hofstede's cultural dimensions at the individual level with adequate reliability and psychometric validity. This approach allows for capturing each individual's personal cultural orientation, recognizing that subcultures coexist within the same country (Taras et al., 2023). The availability of individualized measures such as CVSCALE has facilitated more nuanced studies on how cultural values predict specific attitudes and behaviors of individuals, beyond national generalizations.

Several studies demonstrate the association between of cultural dimensions in health contexts. For example, Chang and Wu (2023) have found a significant correlation between Hofstede's national cultural scores and various indicators of the COVID-19 pandemic in regions of the world, suggesting that cultural values, such as individualism or power distance, modulated the collective response to health measures and compliance with prevention rules Matus (2021). In their study, they mention that culture emerges as an explanatory factor for differences in national health outcomes, with reports indicating that Hofstede's six dimensions, taken together, can predict substantial percentages of the variation in indicators such as life expectancy or healthcare spending. More specifically, Simha et al. (2023) acknowledges that culture shapes sexual practices; for example, more collectivist societies are associated with higher levels of stigma toward HIV, while more egalitarian and performance-oriented societies are associated with less stigma.

### 2.2 Theory of planned behavior

The Theory of Planned Behavior (TPB) proposed by Ajzen (1991) posits that behavioral intention is a widely used socio-psychological framework for explaining the intention to perform a behavior. This theory posits that the best predictor of behavior is behavioral intention, which in turn is determined by three constructs: attitude toward the behavior, subjective norms, and perceived behavioral control (Sun and Moon, 2024). These constructs allow us to predict with high accuracy the intentions to carry out various behaviors, and these intentions, in themselves, explain a considerable proportion of the variability observed in actual behavior (Ajzen, 1991). Thus, the TPB has become one of the most validated and applied theories for understanding behaviors in different domains, including prominently the field of public health (Hagger and Hamilton, 2025).

Within the TPB, the attitude toward the behavior refers to the individual's personal evaluation of performing that action; meanwhile, the subjective norm refers to the perceived social pressure or beliefs about the expectations of significant people regarding whether or not one carries out the behavior in question (Sun and Moon, 2024). These two factors tend to be deeply linked to the individual's cultural and social background; for example, in more collectivist societies, subjective norms may have a greater predictive weight on the formation of intention (Tamuliene et al., 2024). The third construct, perceived behavioral control, refers to the person's assessment of the ease or difficulty of performing the behavior, including the perception of having the necessary capabilities, resources, or opportunities (Ajzen, 1991). While perceived control remains important, it tends to show less cultural variation than attitudes and norms in its relationship to behavioral intention (Fischer and Karl, 2025), this is because perceived control depends largely on objective or individual conditions, such as knowledge, skills, and availability of resources, rather than on shared cultural values (Godin and Kok, 1996). Consequently, when studying behaviors in different cultural environments, the role of attitudes and subjective norms, which reflect culturally learned social values and norms, is usually emphasized, while perceived control is recognized as important at the level of personal efficacy but does not vary systematically across cultures (Fischer and Karl, 2025).

The application of TPB in health behavior has consistently shown its usefulness in predicting and understanding preventive behaviors Capasso et al. (2024) demonstrated that the TPB. It was effective in explaining and predicting vaccination intention. This supports its usefulness in understanding preventive health behaviors aimed at controlling an infectious disease. More specifically, in the study of Derakhshan et al. (2025) determined that the educational intervention significantly improved the behavioral intentions to perform HIV testing on healthcare personnel, mediated by positive changes in the theory's three constructs: attitude toward behavior (attitude), subjective norms, and perceived behavioral control. These studies validate the TPB as a robust theoretical framework for designing interventions and understanding the psychosocial dynamics of health behavior.

## 3 Hypothesis development

### 3.1 Cultural dimensions as predictors of attitude

Cultural characteristics significantly associated with individual attitudes toward preventive health behaviors (Nguyen et al., 2024). A strong long-term orientation, understood as a cultural vision focused on future goals, tends to promote more favorable attitudes toward prevention, by highlighting the sustained benefits of health protection over time (Alkire et al., 2023; Cabrera et al., 2009). People with a high aversion to uncertainty have a greater preference for reducing risks and reacting in a more protective way, which includes having attitudes where the risk is clearer and preventive actions are structural and evident (Appel et al., 2024). Likewise, cultures of indulgence, as opposed to those of restriction, characterized by the free gratification of needs for enjoyment and a marked collective optimism, influence people's positive attitudes toward pleasant experiences related to personal satisfaction. Therefore, health prevention can be positively valued as a strategy to maintain conditions that allow for long-term enjoyment of life (Heydari et al., 2025).

In the field of health, Matus (2021). The study found that cultural indulgence correlates with positive attitudes toward prevention. In contrast, contexts of high cultural masculinity prioritize values such as competitiveness, self-sufficiency, and strength, which can discourage the expression of vulnerability and seeking help for health issues. In this context, preventive behaviors may be perceived as signs of weakness or lack of control, leading to a lower perception of risk and less favorable attitudes toward HIV prevention (Mwansa et al., 2025). This evidence suggests that cultural values such as long-term orientation, risk aversion, indulgence, and masculinity provide favorable attitudes toward the intention to prevent illness for one's wellbeing; therefore, the following hypotheses are proposed:

H1: Long-term orientation is positively associated with attitudes toward HIV prevention.

H2. Masculinity negatively associated with attitudes toward HIV prevention.

H3. Uncertainty aversion is positively associated with attitudes toward HIV prevention.

H4. Indulgence is positively associated with attitudes toward HIV prevention.

### 3.2 Cultural dimensions as determinants of subjective norms

Similarly, cultural values modulate the weight of subjective norms in preventive intentions. In collectivist societies, people attach great importance to the opinions and norms of their reference group; consequently, social pressure on behavioral intentions tends to be stronger in collectivist cultures than in individualistic ones (Morren and Grinstein, 2021). In other words, social pressure to adopt preventive behaviors is often a more powerful determinant of behavior in collectivist environments, where conformity to the group is a core value (Alkire et al., 2023).

On the other hand, a high power distance intensifies deference to authority and hierarchies, which can reinforce norms originating from figures of power. Dong et al. (2022) suggest that in cultures with high power distance, people show greater motivation to comply with the expectations of their superiors or society, thus amplifying the effect of perceived social norms on their behavior. Finally, Zheng et al. (2025) believe that highly permissive cultures predict proactivity, which conceptually may be linked to more open subjective norms, energetic communication, and a positive behavioral predisposition toward social or economic situations. In contrast, restrictive cultures tend to impose strict norms that stifle free discussion, which could limit the presence of subjective norms favorable to HIV prevention. Overall, the literature shows that a culture that values collective cohesion, respect for legitimate authority, and the free expression of wellbeing tends to generate a normative climate more conducive to adopting preventive behaviors; therefore, the following hypotheses are proposed.

H5: Collectivism has is positively associated with on subjective norms.

H6. Power distance is positively associated with subjective norms.

H7. Indulgence has is positively associated with on subjective norms.

### 3.3 Perceived behavioral control determines intentional behavior

Within the Theory of Planned Behavior (TPB), perceived behavioral control (PBC) is one of the main determinants of behavioral intention, as it reflects people's perception of their ability to adopt preventive behavior. Supporting this is the study by Tena et al. (2024), who found that perceived behavioral control was the factor most strongly associated with the intention to use preconception care. In the context of HIV, and considering that this research involves healthcare workers, it is worth highlighting the study by Derakhshan et al. (2025), who demonstrated that a TCP-based intervention strengthened perceived behavioral control and significantly increased the intention to get tested for HIV, while Zeleke et al. (2024) found that greater perceived behavioral control is associated with a greater intention to use HIV self-testing among young people. For their part, Kyynärsalmi et al. (2025) note that distance from power in healthcare settings influences decision-making autonomy, while Leong et al. (2022) found that collectivism steers individual health decisions toward a group's norms and expectations. Based on this, it can be argued that in Amazonian indigenous contexts, where cultural dimensions are highly significant, perceived behavioral control may be influenced by distance from power and collectivism in relation to HIV prevention. Although distance from power and collectivism may limit individual autonomy, these factors may operate differently in the context of indigenous communities. Trust in local authorities and social cohesion can strengthen the perception of control by providing guidance, support, and social validation for HIV-preventive behaviors. Therefore, based on these findings, the following hypotheses are proposed:

H8. Perceived behavioral is positively associated with the intention to prevent HIV.

H9. The distance to power is positively associated with the Perceived behavioral

H10. The collectivism is positively associated with the Perceived behavioral.

### 3.4 Attitude and subjective norms as determinants of behavioral intention

Both the attitude and subjective norms It is identified as a direct determinant of the intention to engage in preventive behavior (Aschwanden et al., 2021). Li and Li (2023), this demonstrates that positive attitudes toward HIV prevention and self-efficacy showed direct and significant effects on the intention to adopt preventive behaviors; that is, people with more favorable attitudes toward the use of preventive measures, such as the use of contraceptives, showed a greater willingness to prevent HIV. Furthermore, Nursinah and Muslimin (2023) found that positive attitudes toward HIV prevention are significantly related to preventive behaviors in an Indonesian community. On the other hand, subjective norms are also a determining factor in intention. Li and Li (2023) found that social pressures and expectations from the environment increase the likelihood of adopting preventive behaviors, showing the importance of social support in HIV prevention. Overall, improving attitudes toward HIV prevention and fostering a social environment through subjective norms are factors that increase the intention to prevent HIV; therefore, the following hypotheses are proposed:

H11. Attitude is positively associated with the intention to prevent HIV.

H12. Subjective norms is positively associated with the intention to prevent HIV.

## 4 Methodology

### 4.1 Sample

To evaluate the proposed research model, a structured questionnaire in paper format was administered to a sample of 497 individuals belonging to the Awajún and Wampís indigenous peoples, both of which belong to the Jívaro language family. These groups were selected because they constitute the largest ethnic groups in the Peruvian Amazon. Santos et al. (2024), representing a case of particular relevance for the study of preventive behaviors against HIV in indigenous contexts. The participants came from various communities, including Wawas, Pakui, Imaza, and Nieva, among others, located in the provinces of Bagua and Condorcanqui, in the Amazonas region.

The study employed a non-probability consecutive sampling strategy, recruiting participants in community spaces such as town squares, community meetings, and agricultural areas. The inclusion criteria required that participants be between 18 and 70 years of age and have resided in the community for at least 1 year. To ensure a climate of trust, recruitment was facilitated by local teachers and coordinated with traditional leaders, who served as cultural mediators and interviewers. As detailed in the data cleaning process, after excluding incomplete responses and outliers, a final sample of 467 participants was used for the analysis.

### 4.2 Data collection and measurement scales

A structured questionnaire was designed incorporating scales previously validated in the literature, adapted to the objectives of this research. To ensure data reliability in an intercultural context, the instrument was translated into the Awajún and Wampís languages by a certified translator registered with the National Registry of Translators and Interpreters of Indigenous Languages of the Ministry of Culture of Peru. Subsequently, a member of the research team, who is a fluent speaker of the indigenous languages, conducted an expert review of the items to ensure they were culturally appropriate and aligned with the local worldview. Finally, the translated items were reviewed with the bilingual teachers who administered the survey to standardize the oral explanations provided to participants.

The questionnaire included sociodemographic variables (community of origin, age, and gender) and used a 5-point Likert scale, with response options ranging from 1 (strongly disagree) to 5 (strongly agree). Cultural dimensions were measured using the theory established by Hofstede (2001), adapted to the individual levelfollowing the approach of Yoo et al. (2011). Variables related to HIV prevention intention were assessed using constructs derived from the Theory of Planned Behavior (Ajzen, 1991), this theory posits that behavioral intention is determined by attitudes, subjective norms, and perceived behavioral control. Appendix 1 presents the specific items used for each construct, the original sources of the scales, and the adaptations made to align them with the cultural context and the study's objectives.

Data collection took place between September and December 2025, through in-person administration of the questionnaire by teachers affiliated with the Teacher Training Program in Intercultural Bilingual Education. These interviewers received prior training on administering the instrument and understanding the content of each item, enabling them to address participants' questions throughout the process. From an initial sample of 497 individuals, those with irregular response patterns, incomplete questionnaires, or outliers identified through preliminary analysis were excluded, resulting in a final sample of 467 participants valid for analysis.

### 4.3 Confirmatory factor analysis of the measurement instrument

The validity and reliability of the questionnaire were assessed using the Amos statistical software. To evaluate construct validity, a confirmatory factor analysis (CFA) was used following the approach of Suhr (2006). The coefficients of determination (*R*^2^) and global fit indices (CFI, TLI, RMSEA, and SRMR) were evaluated. Convergent validity was assessed using composite reliability and variance extraction, as indicated by Bagozzi and Yi (1988). Items with an individual reliability less than 0.50 were excluded (PC2, PC3, PD2, AU2, AU3, C3, C5, MAS4, LTO1, LTO2, I4, ACT3, SN1, SN2, BI1), and the subsequent estimation showed a favorable fit, as shown in Table 1.

**Table 1:** Confirmatory factor analysis.

Constructs or latent variables	Observed variable	Standardized coefficients (*t*-value)	95% CI (LL–UL)	Individual reliability (*R*^2^)	Composite reliability	Variance extracted
Perceived control	PC1	0.699 (^***^)	(0.600–0.800)	0.451	0.660	0.493
	PC4	0.705 (14.331)	(0.609–0.801)	0.501		
Power Distance	PD 1	0.794 (^***^)	(0.702–0.890)	0.631	0.761	0.518
	PD 3	0.764 (13.553)	(0.654–0.874)	0.584		
	PD 4	0.724 (14.123)	(0.624–0.824)	0.589		
Avoidance of uncertainty	AU 1	0.704 (^***^)	(0.605–0.803)	0.501	0.791	0.558
	AU4	0.766 (15.511)	(0.669–0.863)	0.584		
	AU5	0.767 (11.375)	(0.635–0.899)	0.588		
Collectivism	C 1	0.732 (^***^)	(0.633–0.831)	0.536	0.755	0.508
	C 2	0.761 (13.449)	(0.650–0.872)	0.578		
	C 4	0.740 (13.851)	(0.635–0.845)	0.510		
Masculinity	MAS 1	0.816 (^***^)	(0.721–0.912)	0.667	0.744	0.502
	MAS 2	0.771 (14.821)	(0.669–0.873)	0.595		
	MAS 3	0.781 (14.831)	(0.678–0.884)	0.629		
Long-term orientation	LTO 3	0.798 (^***^)	(0.701–0.895)	0.637	0.770	0.530
	LTO 4	0.748 (14.844)	(0.649–0.847)	0.560		
	LTO 5	0.769 (13.561)	(0.658–0.880)	0.510		
Indulgence	I 1	0.793 (^***^)	(0.696–0.890)	0.629	0.753	0.505
	I 2	0.800 (14.065)	(0.689–0.911)	0.640		
	I 3	0.780 (14.925)	(0.678–0.882)	0.629		
Attitude	ACT 1	0.882 (^***^)	(0.800–0.964)	0.777	0.903	0.758
	ACT 2	0.940 (28.081)	(0.874–1.006)	0.883		
	ACT 4	0.782 (21.067)	(0.709–0.855)	0.611		
Subjective norms	SN 3	0.712 (^***^)	(0.613–0.811)	0.508	0.820	0.605
	SN 4	0.809 (14.921)	(0.703–0.915)	0.655		
	SN 5	0.830 (15.174)	(0.726–0.934)	0.688		
Behavioral intention	BI2	0.711 (^***^)	(0.612–0.810)	0.506	0.906	0.558
	BI3	0.757 (15.325)	(0.660–0.854)	0.573		
	BI4	0.774 (15.658)	(0.677–0.871)	0.599		
	BI5	0.769 (15.561)	(0.672–0.866)	0.510		
	BI6	0.766 (15.511)	(0.669–0.863)	0.584		
	BI7	0.742 (15.022)	(0.645–0.839)	0.550		
	BI8	0.707 (14.331)	(0.610–0.804)	0.500		

X^2^ = 1,299.550 df = 450; RMSEA = 0.043; NFI: 0.926; CFI: 0.957. ^***^Indicates the reference indicator whose unstandardized factor loading was fixed to 1.0 for model identification.

Also, the standardized residuals were examined, confirming that the vast majority of the values fall within the acceptable range of ±2.58, indicating that there are no significant discrepancies between the observed and estimated covariance matrices.

Discriminant validity was assessed using the criterion Fornell and Larcker (1981). Table 2 shows that, in all cases the square root of the AVE of each construct was higher than its correlations with the other constructs, which confirms an adequate discriminant validity of the model (Table 3).

**Table 2:** Discriminant validity matrix.

Construct	I	BI	ACT	PC	SN	PD	AU	C	MAS	LTO
I	0.711									
BI	0.433	0.747								
ACT	0.063	0.248	0.870							
PC	0.686	0.498	−0.142	0.692						
SN	0.112	0.308	0.715	−0.019	0.778					
PD	0.072	−0.258	0.112	0.280	0.244	0.720				
AU	0.349	0.342	−0.083	0.520	−0.112	0.184	0.802			
C	0.443	0.500	−0.107	0.557	0.098	0.089	0.661	0.713		
MAS	0.155	−0.183	0.318	0.313	0.422	0.584	0.251	0.099	0.709	
LTO	0.480	0.367	0.000	0.503	−0.125	−0.005	0.791	0.465	0.128	0.798

MAS, masculinity; ACT, attitude; BI, intention; I, indulgence; LTO, long-term orientation; C, collectivism; PD, power distance; UA, uncertainty avoidance; SN, social norms. The values on the diagonal represent the square root of the average variance extracted (AVE), while the values off the diagonal correspond to the correlations between the constructs.

**Table 3:** Heterotrait-Monotrait. All calculated HTMT values fall below the threshold of 0.85, ensuring that the model has robust discriminant validity according to contemporary SEM standards.

Construct	MAS	ACT	BI	I	LTO	C	PD	UA	SN
MAS	—								
ACT	0.382	—							
BI	0.245	0.481	—						
I	0.211	0.105	0.512	—					
LTO	0.198	0.089	0.443	0.562	—				
C	0.154	0.092	0.598	0.490	0.612	—			
PD	0.694	0.187	0.284	0.063	0.041	0.082	—		
UA	0.312	0.114	0.395	0.387	0.787	0.771	0.294	—	
SN	0.495	0.711	0.354	0.158	0.162	0.124	0.341	0.142	—

To protect data integrity, we addressed potential method bias through field procedures and statistical checks. From a statistical standpoint, Harman's one-factor test showed that a single factor explains only 17.395% of the variance, well below the 50% threshold. This confirms that common method bias does not compromise our results.

## 5 Results

**Figure 1:**
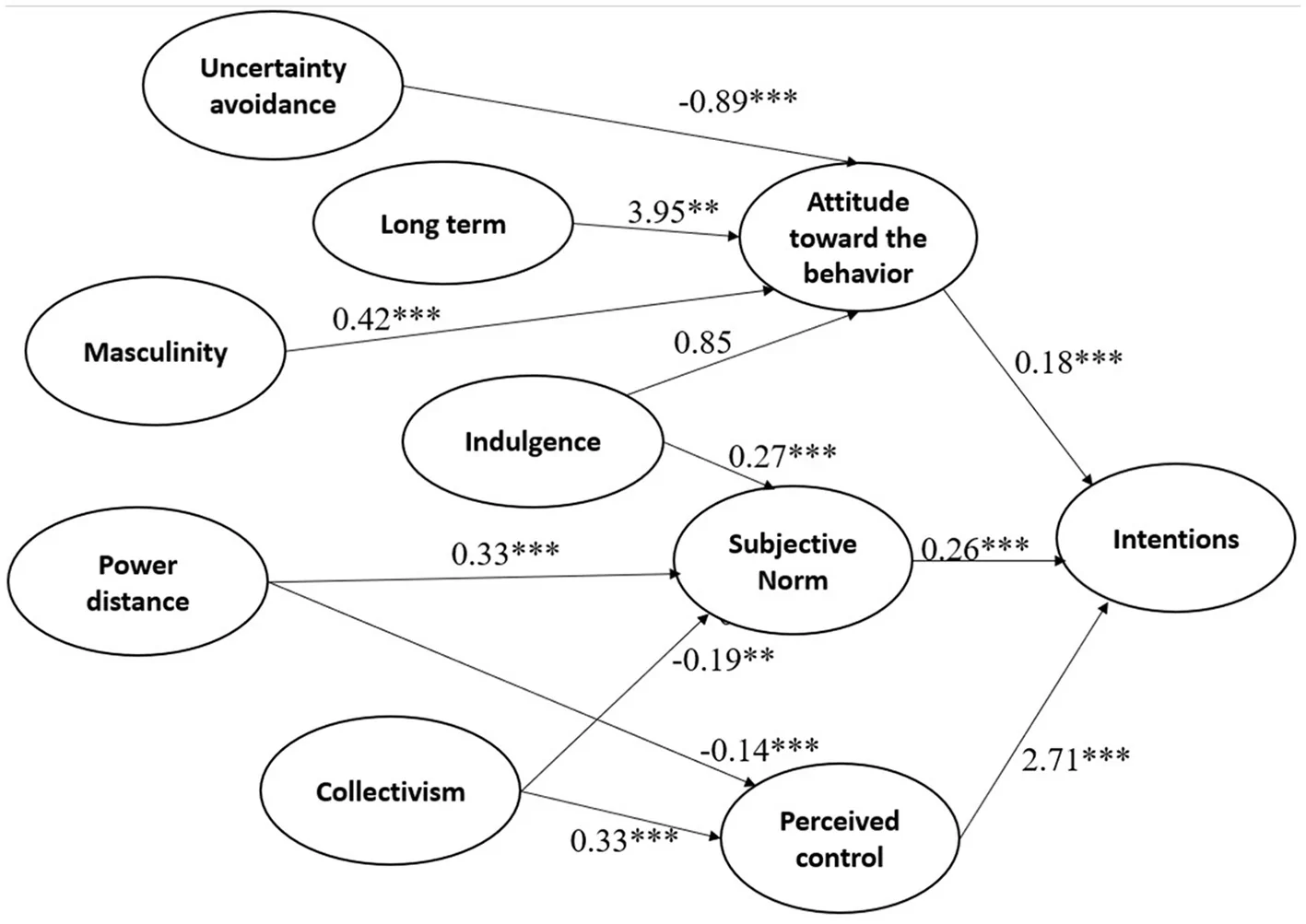
Structural equation modeling. *** = *p* < 0.001; ** = *p* < 0.05. The values shown correspond to standardized regression coefficients (β) estimated using structural equation modeling (SEM), which indicate the magnitude and direction of the associations between the constructs.

The hypotheses were evaluated using a structural equation model (see Figure 1). The model was considered adequate, including a *X*^2^ of 1,661.762, CMIN/DF of 3.551, a DF of 468, an RMSEA of 0.051, and a CFI of 0.927. Among the nine effects evaluated, five showed statistically significant results.

Regarding the cultural dimensions associated with attitudes toward HIV prevention, long-term orientation showed a positive coefficient (β = 0.395^**^) which is consistent with the proposed direction, thus supporting hypothesis H_1_. Masculinity showed a moderate and statistically significant positive association with attitudes toward HIV prevention (β = 0.42^***^); however, this result did not allow for confirmation of hypothesis H_2_ as proposed.

For its part, uncertainty aversion showed a negative coefficient, of high magnitude and statistically significant (β = −0.89^***^). Since the observed direction differs from the proposed one, hypothesis H_3_ was not confirmed. Finally, indulgence showed a positive effect of low magnitude (β = 0.85) without statistical significance, therefore hypothesis H_4_ is not supported.

Regarding subjective norms, collectivism showed a negative coefficient, of low magnitude and not significant, thus not allowing confirmation of hypothesis H_5_. In contrast, power distance showed a positive and statistically significant relationship with subjective norms, with a moderate magnitude (β = 0.33^***^), which confirms hypothesis H_6_. Likewise, indulgence showed a positive and significant effect on subjective norms, of moderate magnitude (β = 0.27^**^), which allowed confirmation of hypothesis H_7_.

Additionally, the power distance did not show a statistically significant relationship with perceived behavioral control in terms of direct effect on intentions, therefore hypothesis H_9_ was not supported. However, collectivism showed a positive and statistically significant relationship with perceived behavioral control (β = 0.33^***^), allowing the confirmation of hypothesis H_10_.

In the final stage of the model, attitude toward HIV prevention showed a positive and statistically significant effect on the intention to prevent HIV (β = 0.18^**^), thus confirming hypothesis H_11_. Meanwhile, subjective norms showed a positive and significant effect on the intention to prevent HIV, with a magnitude greater than that observed for attitude (β = 0.26^***^), allowing the confirmation of hypothesis H_12_. Perceived behavioral control showed a positive and statistically significant relationship with intentions (β = 2.71^***^), which supports hypothesis H_8_.

## 6 Discussion and implications

This study addressed a gap in the literature on HIV prevention by empirically integrating, at the individual level, Hofstede's cultural dimensions with the Theory of Planned Behavior (TPB) in a largely unexplored context: the Awajún and Wampis indigenous communities of the Amazon. While previous studies have shown that culture is associated with health outcomes and preventive behaviors (Chang and Wu, 2023; Matus, 2021), there is a lack of evidence analyzing how individual cultural orientations modulate HIV prevention intentions in specific indigenous contexts.

By employing individualized measures of cultural values of Yoo et al. (2011), the results help overcome the fallacy associated with national averages, allowing us to understand how personal cultural variations are differentially associated with attitudes, subjective norms, and ultimately, the intention to prevent HIV. The results broaden the applicability of the TPB approach in non-Western cultural settings and provide contextualized evidence for the design of culturally sensitive public health strategies in Amazonian territories.

Regarding H_1_, long-term orientation showed a statistically significant effect on attitudes toward HIV prevention. This result suggests that, in the Awajún and Wampis context, individuals who place greater value on future consequences are more likely to develop positive attitudes toward HIV prevention. This may indicate that preventive behaviors are perceived as investments in future health, family wellbeing, and community continuity. In this regard, this finding is consistent with studies conducted in non-indigenous populations, where long-term orientation has indeed been shown to play a significant role in promoting healthy behaviors. Alkire et al. (2023), in South Africa, Mexico, Portugal, and the United States, conceptualize long-term orientation as a cultural value measured at the individual level using the CVSCALE and conclude that it acts as a psychological driver that transforms future-oriented thinking into health-related intentions, thereby improving quality of life. The present study extends this evidence by showing that this cultural orientation also operates as a predictor of health-related attitudes within an indigenous amazonian context. Similarly, Cabrera et al. (2009), in the United States, using an approach based on the Future Time Perspective Inventory in vulnerable populations, observed that a greater future orientation was associated with a lower propensity to engage in behaviors that increase the risk of contracting HIV. Although these studies were conducted in different sociocultural settings, the convergence of the findings suggests that future-oriented values may represent a cross-cultural psychological resource that favors preventive health attitudes. however, the present study contributes new evidence by demonstrating this relationship at the individual cultural level among awajún and wampis communities.

Regarding H_2_, the results revealed a positive and significant effect of masculinity on attitudes toward prevention, contradicting the initial hypothesis. This finding suggests that, in these communities, values associated with cultural masculinity, such as responsibility, strength, and self-sufficiency, may be reinterpreted not as a rejection of prevention, but as an active commitment to caring for one's own health and that of one's immediate environment. In this sense, masculinity could be operating as a framework for social responsibility rather than a barrier (Mwansa et al., 2025). Thus, although certain masculine norms may discourage testing and treatment due to fear of losing status, there are also attitudes that protect masculinity in which health care is viewed as behavior compatible with masculine responsibility and social roles (Odii et al., 2026). This finding invites a reconsideration of one-dimensional interpretations of masculinity in Indigenous contexts. This aligns with the evidence presented by Frisancho et al. (2026), who show that moral decisions among Amazonian Indigenous peoples are guided by responsibilities toward family and community, allowing us to understand preventive behaviors as socially valued. In contrast, in the field of health, researchers have approached masculinity through the lens of gender norms or social beliefs, using instruments other than the CVSCALE. Peterson et al. (2022) in a sample of male college students, finds that traits such as self-reliance can inhibit prevention depending on social validation and the healthcare context. Similarly, (Sedani et al. 2025) report that traditional masculinity has both restrictive and facilitating effects on the intention to use preventive services. This difference could be explained by the relational and communal nature of various Indigenous contexts, where gender roles are linked to collective responsibilities and the wellbeing of the group, rather than to individual logic (Wildcat and Voth, 2023). In this framework, masculinity does not necessarily act as a barrier to prevention, but can be reconfigured as a mechanism for caring for and protecting the immediate environment (Bühler et al., 2021).

Regarding H_3_, uncertainty aversion showed a significant negative effect on attitudes toward prevention, contrary to what had been suggested. This result suggests that a high intolerance of uncertainty could generate avoidance responses to complex and stigmatized threats such as HIV, reducing the willingness to value prevention positively. In contrast, studies such as Appel et al. (2024) approach this construct from a psychological perspective, using the Uncertainty Intolerance Scale which measures cognitive and behavioral responses to doubt and ambiguity. This interpretation is consistent with Nicolás-Martínez et al. (2026), who found that stigma, misconceptions about HIV, and cultural barriers continue to hinder diagnosis, treatment, and the adoption of prevention measures among indigenous peoples in Latin America and the Caribbean. Furthermore, Troccoli et al. (2026) show that stigma and the coexistence of biomedical and traditional practices influence risk perception and the pursuit of care; thus, when multiple interpretations of the disease exist, a greater need to reduce uncertainty may favor avoidance responses rather than a greater willingness toward prevention. From this perspective, it has been found that low tolerance for uncertainty can motivate the adoption of risk-reducing behaviors. This difference can be explained by variations in the environment; in contexts such as Europe, characterized by greater access to information, health services, and a sense of control, uncertainty can trigger responses aimed at reducing risk (Gupta and Rudisill, 2024). In contrast, in indigenous communities, where there are structural limitations in access to health services and higher levels of social vulnerability (Armenta et al., 2021), uncertainty could generate avoidance responses to complex and stigmatized threats such as HIV.

Indulgence did not show a significant effect on attitude, leading to the rejection of H_4_. This result is particularly relevant, as it suggests that, although indulgence is associated with subjective wellbeing and optimism (Hofstede, 2001), however, these values do not necessarily translate into a positive evaluation of HIV prevention at the attitudinal level. In Awajún and Wampis contexts, the orientation toward present wellbeing may be more linked to daily harmony and immediate community equilibrium than to the conscious adoption of specific preventive behaviors, which limits its direct impact on attitude. In contrast, Matus (2021), drawing on Hofstede's cultural dimensions based on national scores, indicates that indulgence is associated with higher levels of subjective wellbeing and quality of life, including indicators such as life expectancy. However, this type of measurement does not necessarily capture how these values translate into specific attitudes toward preventive behaviors in particular contexts.

Regarding subjective norms, collectivism did not show the expected positive effect (H_5_), suggesting that the strong group cohesion characteristic of these communities does not always translate into explicit normative pressures in favor of HIV prevention. This result may reflect the persistence of social silences or taboos surrounding HIV. From this perspective (Simha et al., 2023), drawing on the Global Values Scale, demonstrate that collectivism is associated with higher levels of stigma toward people living with HIV. In this sense, the absence of a positive effect of collectivism on subjective norms suggests that, in contexts where HIV is socially stigmatized, group cohesion does not necessarily promote prevention, but may operate through dynamics of silence or avoidance rather than explicit normativepressure. Conversely, power distance showed a positive and significant effect on subjective norms, confirming (H_6)_. This finding indicates that, in communities where respect for authority is central, the expectations and messages from legitimate figures, such as community leaders or health workers, have a relevant normative weight in the social perception of prevention. This result is consistent with the findings reported by Dong et al. (2022), adapted from Youngdahl et al. (2003) and subjective norm is distinguished between normative beliefs and motivation, following Bock et al. (2006). Their findings show that power distance does not uniformly affect the subjective norm, but rather specifically strengthens individuals' motivation to align with the expectations of authority figures and social references. It is important to note that, within the Awajún and Wampis communities, the association of power distance operates within a community framework, where figures of authority are primarily local—such as the APU—unlike in formal organizational settings, as in the case of Dong et al. (2022).

Consistent with H_7_, the cultural value of indulgence showed a positive and significant effect on subjective norms. This result suggests that, indulgence contribute to creating a more open and communicative social environment where prevention can be socially discussed and normatively accepted. In this sense, indulgence appears to facilitate the circulation of messages and social expectations favorable to prevention, aligning with what was proposed by Zheng et al. (2025).

Regarding perceived behavioral control, power distance did not show a significant effect, therefore H_9_ was not supported. This result suggests that, although respect for authority figures may influence social expectations and subjective norms, it does not necessarily translate into a greater perception of personal capability to perform hiv prevention behaviors. This finding is consistent with the idea that perceived behavioral control reflects individual assessments of resources, skills, and opportunities to perform a behavior (Ajzen, 1991), which may not be directly determined by hierarchical relationships. In indigenous settings, authority structures may provide guidance and social order, but individuals may still perceive hiv prevention as a personal decision requiring specific knowledge, access, and self-efficacy.

In contrast, collectivism showed a positive and significant association with perceived behavioral control, supporting H_10_. This finding suggests that collective orientation can function as a source of psychological and social resources that enhance individuals' perception of control over preventive behaviors. In the Awjún and Wampis communities, where social relationships and reciprocal support are central elements of community life, individuals may feel more capable of adopting hiv prevention actions when these behaviors are supported by their social networks. This result expands previous evidence on collectivism and health behavior by showing that collective values do not only operate through social norms but may also strengthen perceived capability to act. This finding is consistent with Leong et al. (2022), who highlight that collectivist orientations shape health decisions through social support and shared responsibility. However, unlike contexts where collectivism may reinforce dependence on the group, in this indigenous context it appears to provide social resources that facilitate personal control over preventive actions.

The results confirm that both attitudes (H_11_) and subjective norms (H_12_) positively and significantly predict the intention to prevent HIV, with the effect of subjective norms being stronger. This pattern is consistent with the TPB (Ajzen, 1991). In particular, studies by Li and Li (2023) and Tamuliene et al. (2024) show that the intention to prevent HIV depends not only on favorable individual assessments but, to a greater extent, on the normative weight and expectations of the social environment. However, it is important to note that these studies use different instruments to measure attitudes and subjective norms based on scales adapted from Green (1995) and Ross and McLaws (1992), suggesting that, while the results are convergent at the theoretical level, there are differences in the operationalization of the constructs. In the case of the Awajún and Wampis communities, the greater predictive weight of subjective norms can be explained by the central role of social relationships and collective dynamics in decision-making. In these contexts, individual decisions are more closely tied to the expectations of significant others and community leaders (Fernandez et al., 2023).

From a theoretical perspective, the results reinforce the usefulness of integrating the TPB with cultural dimensions measured at the individual level, showing that cultural values do not operate uniformly across all constructs of the model. In particular, indulgence emerges as a factor that has a clearer association with the normative than on the attitudinal level, making a novel contribution to the literature on culture and health behavior. Likewise, the findings challenge linear assumptions about masculinity and uncertainty, demonstrating that their impact depends on the specific sociocultural context. This suggests that Hofstede's dimensions should be interpreted as flexible and relational frameworks, rather than as universal determinants, especially in Indigenous populations with their own social and territorial structures.

From an applied perspective, the results suggest that HIV prevention strategies in Awajún and Wampis communities should prioritize strengthening positive subjective norms, leveraging the influence of legitimate authorities and community leaders such as the APU (Association of Community Leaders). Health communication could benefit from approaches that legitimize prevention as a socially approved practice consistent with community values. Furthermore, given that leniency contributes to a more favorable normative climate, preventive messages could be framed in terms of collective wellbeing and the continuity of community life, rather than in discourses focused exclusively on risk. For policymakers and health professionals, these findings underscore the importance of designing culturally situated interventions that acknowledge the normative and authority dynamics present in the territory.

This study has some limitations that should be considered. First, the data collection process presented operational challenges stemming from the sociocultural characteristics of the study population. In particular, the heterogeneous levels of literacy necessitated the use of interviewer-administered surveys, which required participants to explain the items orally and in context. While this procedure required more time and resources, it ensured participants understood the instruments and effectively included those who might otherwise have been excluded, thus strengthening the study's internal validity and cultural relevance. Furthermore, future research could complement the quantitative approach with qualitative methods that delve deeper into the local meanings associated with prevention and health. Finally, extending the study to other Indigenous communities would allow for an assessment of the generalizability of the findings and a more detailed examination of the role of the territorial context in the relationship between culture and HIV prevention. Additionally, the study did not systematically track individual refusal rates during the field collection phase. While a rigorous data-cleaning process was applied, resulting in the exclusion of 6% of the initial sample due to incompleteness.

Furthermore, the model did not incorporate sociodemographic control variables that could influence both cultural values and the psychosocial determinants of prevention intent. This limitation should be considered when interpreting the results, as research such as that by Valenzuela-Oré et al. (2023) has shown that factors such as occupation and socioeconomic conditions are associated with HIV-related behaviors. Furthermore, future research could complement the quantitative approach with qualitative methods that delve deeper into the local meanings associated with prevention and health. Finally, extending the study to other Indigenous communities would allow for an assessment of the generalizability of the findings and a more detailed examination of the role of territorial context in the relationship between culture and HIV prevention.

## 7 Conclusions

The study analyzed the association between of Hofstede's individual cultural dimensions on the psychosocial determinants of HIV prevention intention, integrating this cultural framework with the Theory of Planned Behavior in Awajún and Wampis native communities. The results confirm that individual cultural values play a significant role associated with attitudes, subjective norms, and ultimately, preventive intention, underscoring the importance of considering the specific sociocultural context when designing public health interventions.

Regarding attitudes toward HIV prevention, the findings show that not all cultural dimensions operate in the theoretically expected direction. Long-term orientation and indulgence did not show significant effects, suggesting that, in these territorial contexts, valuing future benefits and emphasizing subjective wellbeing do not necessarily translate into favorable attitudinal evaluations of specific preventive behaviors. Similarly, uncertainty aversion had a negative effect, indicating that greater intolerance of ambiguity can hinder the adoption of preventive attitudes toward a complex and socially sensitive disease like HIV. In contrast, masculinity showed a positive and significant effect on attitudes, suggesting that, in the communities studied, values associated with strength and responsibility can be redefined as motivators for health care, challenging traditional interpretations that associate masculinity with lower prevention rates.

Regarding subjective norms, the results highlight that the social and normative environment is a central factor in HIV prevention. Power distance and leniency were positively associated with favorable subjective norms, underscoring the importance of legitimate authority and open social climates for building collective expectations around prevention. In contrast, collectivism did not show a significant effect, suggesting that social cohesion alone does not guarantee the existence of explicit norms that promote preventive behaviors, especially when silence or stigma associated with HIV persists.

In accordance with the Theory of Planned Behavior, both attitudes and subjective norms positively and significantly predictors the intention to prevent HIV, with the effect of subjective norms being more pronounced. This finding highlights that, in community contexts such as those of the Awajún and Wampis, preventive intention is constructed primarily from shared normative frameworks and the perception of social approval, rather than solely from individual assessments.

From a public policy perspective, the findings support the importance of integrating the cultural dimension as a central component in public health strategies targeting Indigenous populations. Recognizing cultural diversity and the social mechanisms that predictspreventive intent can guide the development of more equitable, inclusive, and contextualized policies, fostering more effective responses to HIV.

## Data Availability

The original contributions presented in the study are included in the article/Supplementary material, further inquiries can be directed to the corresponding author.
