# Supplementary material for: Cultural values and HIV prevention intentions: integrating Hofstede's dimensions and the theory of planned behavior in Amazonian Indigenous communities
Source: Front Psychol. 2026 Aug 28;17:1789791. doi: 10.3389/fpsyg.2026.1789791 (PMC13561805; doi:10.3389/fpsyg.2026.1789791)
Supplement: Supplementary file 1 [file Data_Sheet_1.docx]

**Supplementary Material**

**Table S1. Dimensions in Awajun and English**

| **Dimension** | | **Awajun** | **English** |  |
| --- | --- | --- | --- | --- |
| Power Distance | | Amina batsatkamugminish, Pamukush nigkikish dutikattajai, tumainkaik tikish aindaun iniskesh (ijunbau nuniashkush batsatkamunmaya ijuntuja takatainash) | In your community, the APU should make decisions on its own, without consulting others (such as when to hold the assembly or when to organize community work) |  |
|  |  | Pamukuk jutikaipa tubaitsui batsatkamunmaya aidaunak wajii dutikmain aidaunak. (nugkash wajuk ayamjumainaita nuniashkush tikish batsatkamunmaya tainakuish) | The APU should avoid consulting extensively with community members when making important decisions (such as defending the territory or dealing with outsiders) |  |
|  |  | Pamukuk uyumamaitsui mamiksa takatai aina nunak, datsaush nuniashkush nuwa aidau aina nuna takataijinak ( wajuk iwainmamainaita apashnumia takat pachisa takaku ijunkuish) | The APU should not assign important tasks to young people or women (such as representing the community at a meeting with a government agency) |  |
|  |  | Apu timbaunak batsatkamunmaya muun aidauk atsa tusan tubaitsui (aja yamajam ajamtin batsatkamunu atinun) | The community members should not contradict what the APU has already decided (such as where to open a new communal farm) |  |
| Avoidance of uncertainty | | Aents dekamin nuniashkush ampijatin ujajatkati betekmas tumainai (wajuk umutaita ampish nuniashkush ajak jaaku ampimatainash) | It is important that the wise man or nurse explains step by step (for example, how to take some medicine or medicinal plant) |  |
|  |  | Pamuk tambaush umimainkaitam batsatkamunmash (wetatji namak maku majanu) | In your community, you must obey what the authority (APU) says in the assembly (such as when everyone should go fishing in the river) |  |
|  |  | wagka pegkejaita ijunku takat umiamush, wagka tamaki maki makichik takat umiktin (papi augtai nuniashkush ampimatai kuitambau yapajintsa) | The assembly rules are important because they state what is expected of each person (such as taking turns looking after the school or the health post). |  |
|  |  | Batsatkamunmayan yainin ainawai makichik takatan mamikig numak, jimajan apatkanuk dewakaig tusa (namak nijamu nuniashkush tayu juwaamu) | Community agreements help ensure everyone does the same thing and avoids confusion (like when to do communal fishing, or go to the tayo harvest) |  |
|  |  | Shiig pegkejai wajuk takastatji nunu pachisa ujaniamu (maki makichik ujaniamu dewakaig tusa wajuk takat takastatji nunu takamu batsatkamunum). | It is important that there are always clear instructions and directions for the work (for example, in community work, it should be clearly stated what each person does to avoid mistakes). |  |
| Collectivism | | Wii tikichinum weta tambau akuish, yammainaitjai mina patajun nuniashkumush wii batsatkamunmayan (ajajun ukuakun wemainaitjai ijunbaunum) | Even if I want something else, I must support my family and community (like leaving my farm to go to the community meeting). |  |
|  |  | Mina ajajui bakichik tsawan menkaekun, wemainaitjai batsatkamunum takat akuig. | I must participate in the communal work, even if I lose a day of work on my farm. |  |
|  |  | Yaimbau batsatkamunum akug shiig pegkejai, wii juwashkunush (yaimbau bakau pachis nuniashkush namak tagkumtan) | It is more important that the community receives a benefit, even if I don't gain much (like when a cocoa project or fish farm arrives for everyone). |  |
|  |  | Mina pataan nuniashkush mina ijagdaitaig yainkata takuig yaimanaitjai, yawejan nuniashkush tikish takat ajutkuishkam (chigkim iitamu nuniashkunush aja takamukesh) | When my family or neighbors ask me for help, I must support them, even if I am tired or busy (like going to carry firewood or helping in the neighbor's farm). |  |
|  |  | Tuke yaimkauk amainaitjai batsatkamunmaya, ijunainak dita mina atsumjuinashkuig | One must always maintain loyalty to the community, even if one does not agree with what was decided in the assembly. |  |
| Masculinity | | Batsatkamunumaya Aishmag aidau jinki ausatnume nuniaskush takastinme. | It is more important for men to go out to study or work outside the community than for women. |  |
|  |  | Aishmagkuk iwagmainai utugchatnak buken, nuwak anentaimas (wajuk uchi augmainaita) | Men solve problems by thinking with their heads, and women with their hearts (like when deciding whether a child should go away to study or stay). |  |
|  |  | Utugchat najamin dutikmainchau aunash iima aishmagkeash dutikmainait t (numi muun ajamu nuniashkush jega jegambau) | To solve difficult problems, only men can use strength (such as cutting down large trees or building a house). |  |
|  |  | Aishman, nuwa nagkaesau umiamush awai takat (kuntin maut nuniashkush jega jegambau) | There are jobs that men do better than women (like hunting or building a house) |  |
| Long-term orientation | | Shig pegkejai ukusa ajakmasa paampa, yujumak ainiashkush wee wagkatamak yuwamu tsawan utuchat minimainanuwi. | It is important to store part of the banana, cassava, or salt harvest for when times of scarcity come. |  |
|  |  | Tikish wakegainashkuish waitka segamainaitjai (emamainai bakau pachisa takamu, tikish nagkama emainashkuish) | It is important to keep insisting, even if others don't want to (like continuing a cocoa project, even if some don't support it at the beginning). |  |
|  |  | juka pegkejai ajutjamain agkag nuwigtu tuk atinanu iina pujutjin | It is important to have peace and stability in life (such as securing the farm and ensuring the children go to school) |  |
|  |  | Emtiksa anentaimjamu amainai waji ajattaji ajanmash nunu(wajuti ajamainata yujumak ajatnush nuniashkush shaakesh) | It's good to plan ahead what to plant on the farm (for example, deciding when to clear land for cassava or corn) |  |
|  |  | Senchi takamainai atak iina uchiji ajiti takuik (wajuk yaimainaita iina uchiji unuimaju atii takuish) | We must work hard today so that our children will be better off in the future (such as striving to help them become professionals). |  |
| Indulgence | | Tsawan agkan sumamainai ayambau nuniashkuish iina kumpajijai pujamu) | It is important to have free time to rest or be with friends (such as going swimming in the river or chatting in the community center) |  |
|  |  | Agkan takamainai, timbau umiaku wekaetsuk (wii wakegaknuk wemainairjai kuntinu makun nuniashkunush pata ijagkun tikish batsatkamunum | It's good to do things freely, without so many rules (like deciding when to go hunting or visit family in another community). |  |
|  |  | Ii wakegamu wekaemaina ima takatnumkechu (nijamash umaku,kantamku, nantsemku jistanum) | It's important to enjoy life and not just work (like drinking masato, singing and dancing at parties) |  |
|  |  | Tsawan amainai wekaembau jistanum, wasugkambaunum iina batsatkamujin ( jiistamku batsatkambau akinamujin nuniashkush depetmaktasa tewa wasugkamku) | It's good to have moments of celebration or play in the community (such as on the community anniversary or in football championships) |  |
| Attitude | Ampimatainum preservativo sukagtambauk pegkejai jata jukig tusa. | | Using the condoms provided by the health center is a good way to avoid getting HIV. | |
|  | Tuke iina iiyashi tsaniakuik nuwajaik apugmainai jata jukig tusa apugtai, iina uchiji, pataji kuitamkami tusa. | | Using condoms in a relationship is a way to protect the family and children. | |
|  | Batsatkamunum jamamtuchu pujusta takuik antumainai jata pachis ujajatbau. | | Listening to talks about HIV is helpful for living a healthy life in the community. | |
|  | Tikish dushikagtus dijatuinakuish, jata jukig tusa apugtaik apugmainai nuwajai tsaniakuik. | | Although some may scoff, I think it's right to use protection in a relationship to avoid disease. | |
| Subjective norms | Jata VIH tuinanauish tunchish ayautkagtumaina tuinawai batsatkamunmayag. | | In my community, some believe that HIV can come from witchcraft or bad air. | |
|  | Mina kumpag tuinawai jata jukig tusa apugtaik, apugchakuish apugmaitsui ampimatainmayak. | | My friends think that it's not always necessary to use the condom that's the best. | |
|  | Mina duwajuk kuitamjumkati tusa tujutui, niijai pujau asamtai. | | My partner expects me to take care of myself when we are together. | |
|  | Pamukuk tukagtawai kuitamjumkatajum jata jukijum tusa VIH,aentsuk ajantus antuinawai. | | When the APU recommends protecting oneself to avoid diseases like HIV, people respect it. | |
|  | Dekamin, pamuk, kuitamjumkatajum tujamji, tutai kushat antujuinawai. | | When the wise man or leader advises taking care, many listen. | |
| Perception of control | Jata jukig tusa apugtaik kakajus ikambaitsui ampimatainmak sujamunmashkam. | | Sometimes it is difficult to get protection at the post office or the community store. | |
|  | Kuashat tuinawai datsantai Jata jukig tusa apugtaik segambauk kuashat batsatbaunum . | | For many, it is shameful to ask for protection in public. | |
|  | Wika kuitamjamkatasan wakegajai, wika ikamainai tajai ampi sujutainum nuniashkush ampimatainum,jata jukig tusa kuitamjamtaik. | | If I want to take care of myself, I know I can get a condom at the pharmacy or the health center. | |
|  | Aents nijamchin umajuk nuniashkush tikich umutain umajuk, kakajus anentaimtsui kuitamjumkatasag. | | When a man has consumed a lot of masato or alcohol, it is more difficult for him to think about protecting himself. | |
| Behavioral intention | Nuwak imanisan sujimantsui jata jukig tusak, wagka tamak aishi tambaun umiau asa. | | Women have little freedom to ask for protection, because their husbands decide. | |
|  | Atak pujustajai maak takunuk, anentaimainaitjai jata VIH jukig tusa apugtain apugmainaitjai | | If I have sex in the future, I'm going to think about using protection so I don't get HIV. | |
|  | Apu jaata pachis VIH untsumtainum untsumkuig antugkatin atajai. | | If I hear a radio or APU message about HIV, I will follow the recommendation. | |
|  | Duwanjai chichastajai wajuk kuitamjamainaita jata jukig tusanush. | | If I have a partner, I will talk to her about how to protect ourselves from HIV. | |
|  | Ampimatainum jata jukim tusa apugtain sukagtakuig jukitjai nunikan ukuktajai atak atsumakun apusatin asan. | | If the post offers protection, I'll accept it and keep it for when I need it. | |
|  | Kuitamjumkatasan wekegajai jata achimakaig tusan VIH tikichin utsukaig tusan, | | In the future, I want to take care of myself so that I don't get HIV or infect others. | |
|  | Utugchata itawai VIH patagjai ,mina apajun, duwajun uchig aindaun jata ajutkagtambauk. | | Having HIV brings many problems to my family, especially to our parents, partner, and children. | |
|  | Ii puyatjumkashbaunum Tikish jata VIH utsujatuk umimainai batsatkamunum chicham umikbaun. | | Infecting others with HIV is an irresponsible act that must be regulated in the community. | |
